# Developing Risk Assessment Items of Treatment Interruption Among Vietnamese Patients with Tuberculosis in Japanese DOTS—A Quantitative and Qualitative Survey Using the Delphi Method

**DOI:** 10.3390/nursrep14040240

**Published:** 2024-11-01

**Authors:** Reiko Mori, Kae Shiratani

**Affiliations:** 1 Faculty of Nursing Department of Nursing, Nihon Fukushi University, Tokai 477-0031, Japan; 2 School of Nursing, The Jikei University, Tokyo 182-8570, Japan; k-shiratani@jikei.ac.jp

**Keywords:** directly observed treatment short-course, public health nurses, risk assessment items, treatment interruption, tuberculosis, Vietnamese patients with TB

## Abstract

Background: The number of foreign-born patients with tuberculosis (TB) has been increasing in Japan, and the number of Vietnamese patients was the highest in 2019. Tuberculosis (TB) is the second leading cause of death from infectious diseases after coronavirus disease-2019 (COVID-19). As the prevalence of TB varies widely globally, measures must be tailored to local characteristics. The Directly Observed Treatment (DOTS) short-course was introduced by the World Health Organization as a global strategy to overcome these challenges. The purpose of this study is to develop an original risk assessment for treatment interruption for Vietnamese patients with TB to be used in Directly Observed Treatment (DOTS), a tuberculosis control measure. Methods: The researchers adopted the Delphi method. Public health nurses of mid-career or above (*n* = 15) who had conducted DOTS for several Vietnamese patients with TB were selected and surveyed about the content and surface validities of the draft risk assessment items for treatment interruption. The survey was conducted three times. The quantification of content validity and the review and modification of responses regarding each item were analyzed by the researchers. Results: The results identified the following risk categories: “physical characteristics”, “background of life during residence”, “treatment environment”, “understanding tuberculosis and disease acceptance”, and “cultural and value trends”. Conclusions: The results suggest the need to provide support for tuberculosis recovery from various perspectives, including the living environment of Vietnamese patients with TB, their social environment in Japan, and the culture and values of their country of birth and upbringing.

## 1. Introduction

Tuberculosis (TB) remains a leading cause of death worldwide, ranking 13th in 2021, and the second leading cause of death from infectious diseases after coronavirus disease 2019 (COVID-19). With the onset of COVID-19 and national conflicts having a detrimental impact on access to diagnosis and treatment and the rising burden of TB, the progress toward global targets has been slowed down [1]. Globally, more than 10 million new cases of TB and approximately 1.6 million (including HIV co-infection) deaths from TB were reported in 2021 [1]. While the disease is treatable and preventable, resistant TB remains a problem, with the amount of Multidrug-Resistant TB/Rifampicin-Resistant TB cases increasing by 3.1%, from an estimated 437,000 in 2020 to 450,000 in 2021 [2].

The same applies for resistant TB in Japan, where 78.1% of newly registered pulmonary TB culture-positive patients in 2022 were reported to have drug susceptibility test results (susceptible to both isoniazid and rifampicin); however, this had not yet reached 80% [3]. The most effective way to prevent the spread of resistant TB is to ensure that the patients complete their treatment. As the prevalence of TB varies widely across countries and regions, measures must be tailored to local characteristics. The Directly Observed Treatment short-course (DOTS) was introduced by the World Health Organization (WHO) as a global strategy to overcome these challenges more than 30 years ago [4,5].

In Japan, the TB incidence rate was 8.2 in 2022 (incidence rate per 100,000 people), which has been decreasing annually. On the other hand, the number of foreign-born patients with TB is increasing, which is an urgent issue in Japan. From 2019 to 2021, the number of foreign-born patients with TB patients decreased due to COVID-19 entry restrictions, but the percentage of patients with TB increased from 11.4% in the previous year to 11.9% in 2022. Regarding foreign-born patients with TB in Japan, the major countries of origin are in Southeast Asia, including the Philippines and Vietnam. In particular, the incidence of TB cases among Vietnamese individuals has continued to increase over the past five years [6]. Vietnam is among the top 30 high-burden TB countries worldwide, and the Beijing TB cluster—which is highly infectious and prone to acquiring drug resistance—is reported to be widespread in Vietnam [1,7]. The main aim of Vietnamese residents in Japan is to work or study. According to the Report on the Employment Status of Foreign Workers in Japan, Vietnam immigrants accounted for 25.4% of all foreign workers—the largest in 2022 [8]. Additionally, among foreigners who wish to study abroad in Japan, Vietnamese students are second only to Chinese students [9]. Considering these numbers, Japan needs to provide appropriate treatment to Vietnamese patients with TB.

In Japan, “The Japanese Version of DOTS” is a basic policy supporting TB patients. This system is a Japanese version of the strategy for TB suppression proposed by WHO DOTS; it has been developed at public health centers across Japan, and is mainly led by public health nurses [10]. The Japanese version of DOTS consists of two stages, namely Hospital DOTS and community DOTS. Hospital DOTS is a system in which medical personnel collaborate with each other during hospitalization, which is required when there is a sputum smear-positive result or when TB bacilli have not been smear-negative. In contrast, community DOTS is a system that targets patients who are treated on an outpatient basis, and where health centers play a central role in supporting recuperating patients. The public health center formulates an individual patient support plan using the risk assessment form for all recuperating TB patients. The risk assessment items used are prepared at each public health center, with reference to the factors responsible for causing treatment interruption of TB patients [11]. Many public health centers have original assessment forms for Japanese tuberculosis patients—but none for foreigners—and use these forms instead [12,13,14,15]. As the number of foreign TB patients continues to increase, public health nurses in charge of DOTS continue to struggle with creating a DOTS plan and deal with individual patients [16,17]. To promote appropriate support for tuberculosis recovery for foreign patients in community DOTS, it is necessary to conduct accurate patient assessments and develop appropriate DOTS plans for individual patients. In other words, it is necessary to develop risk assessment items that fully consider the characteristics of ethnicity, culture, customs, and ways of perceiving disease that differ from those in Japan [18,19,20,21,22]. To provide appropriate treatment support to the increasing number of Vietnamese patients with TB in Japan, risk assessment items specific to Vietnamese patients with TB should be immediately developed.

To improve this situation, our study aimed to develop items that would help assess the risk of treatment interruption among Vietnamese patients with TB in Japan. By accurately assessing this risk of treatment interruption for Vietnamese patients with TB, we can enable better planning and implementation of the support they need. In addition, appropriate treatment continuation and suggestions for the medical and community environment can be offered. Furthermore, our study can be used as a reference for nurses to better understand patient treatment using DOTS for patients with TB from different countries. TB is a disease with chronic respiratory and systemic symptoms. Its treatment requires ongoing chemotherapy with side effects, as well as health checks for several years after the treatment ends. In contemporary nursing practice—where the emphasis is shifting from an acute care model to an integrated care model with a focus on health and social care—the implications of care in the community can be discussed and contribute to the development of nursing practice and the field at large [23,24].

## 2. Materials and Methods

### 2.1. Definition of Terms

DOTS (Directly Observed Treatment short-course): DOTS is a strategy for TB control proposed by WHO and a comprehensive approach that includes five key concepts. DOTS is a method in which a third party (a healthcare worker) checks the medication of patients with TB and monitors their progress to reaching a cured state, aiming to prevent treatment interruptions and the development of resistance to TB bacteria and ensure treatment continuity.

Community DOTS: In community DOTS, health centers formulate an individualized support plan for each TB patient and implement DOTS, with relevant local organizations and occupations acting as “DOTS Partners” and cooperating in formulating the plan. The results of the community DOTS are monitored and evaluated at the DOTS meeting, and the progress of the patient’s treatment is monitored at the cohort meeting. The risk assessment form is used to quantify the patient’s risk of discontinuation of treatment, and the DOTS response level is classified. However, in Japan, there is no standardized risk assessment form for TB patients, as each health center is left to develop one in consideration of regional characteristics and the 10 items listed by the government as treatment interruption risks.

DOTS partners: Public health nurses in charge of Vietnamese patients with TB, medical professionals who support the continuation of treatment of these patients, and welfare personnel are referred to as DOTS partners. In addition, they support Vietnamese patients with TB in their recuperation stage.

### 2.2. Research Design

#### Quantitative and Qualitative Survey Using the Delphi Method

The Delphi method was used in this study. The Delphi method aggregates the opinions and findings of several experts to obtain a unified view. Expert members are asked to respond individually, and the results obtained are fed back to all members, who are then asked to respond individually again. The method aims to repeat this process until the expert opinions generally converge. By providing each participant with feedback on the results of the survey, which have been compiled and revised, each participant can take into account the views of others and re-evaluate their own opinions. By repeating this process, the opinions of the participants unite in their meanings and agreement is reached. By quantifying the results through quantitative analysis, each participant will be able to evaluate them more objectively.

The study collaborators were public health nurses and healthcare workers involved in community DOTS in 48 public health centers in areas with large Vietnamese populations in Japan; public health nurses in their mid-career or higher stages of experience in charge of multiple Vietnamese patients with TB in DOTS, or healthcare workers with equivalent years of experience, were selected. The survey sought opinions on the validity of 28 items in seven categories of the proposed risk assessment items and was conducted 3 times in total.

### 2.3. Selection of Research Collaborators (Participants)

The survey participants were required to have experience being in charge of supporting Vietnamese patients with tuberculosis using the Japanese version of DOTS on multiple occasions, and to have at least intermediate-level experience as public health nurses or equivalent experience as medical professionals. This was to ensure the accuracy of responses. The collaborators were limited to those with at least five years of experience, as the Japanese national career stage for public health nurses indicates that mid-career or above corresponds to the required level [25,26,27]. Due to the limited number of collaborators, the survey was conducted nationwide.

### 2.4. Creation of a Survey to Identify Risk Assessment Items of Treatment Interruption for Vietnamese Patients with TB in Japan

This study was conducted in the following way: Before the study, we developed a standardized risk assessment form for patients with TB in Japan. Meanwhile, we identified risk factors for treatment interruption in Vietnamese patients with TB by conducting interviews, reviewing the literature on the risk of treatment interruption in Vietnamese patients with TB, and developing draft risk assessment items. The draft risk assessment items were surveyed by TB researchers on the validity of their contents.

#### 2.4.1. Standardized Risk Assessment Items in Japanese DOTS

In Japan, no standardized version of the risk assessment form for patients with TB has been developed yet, and the risk assessment forms that consider the characteristics of each region have been used in each public health center. Therefore, we developed a standardized risk assessment form in Japan as a preliminary study. The study area was selected as four prefectures in Tokai, which is geographically located in the middle of Japan and is a miniature region of Japan, including rural areas and designated cities. The results of risk assessment evaluation of TB patients (470 patients) were statistically analyzed to develop a standardized version (draft) of the risk assessment form to be used when creating a DOTS plan. Subsequently, a panel of experts on tuberculosis was convened to review the risk assessment items and weigh each item on the standardized version (draft), and a validated standardized version of the risk assessment form was developed [28].

#### 2.4.2. DOTS Interruption Factors for Vietnamese Patients with TB in Japan

Five medical interpreters who could understand the language of Vietnamese patients, along with healthcare workers in community DOTS, were interviewed to explore the factors that caused these patients to interrupt their treatments. As medical interpreters, they identified factors objectively from a third-party perspective. Medical interpreter professionals use their interpretation skills and medical knowledge to support mutual understanding; however, there are few qualified medical interpreters in Japan. As there were also few medical interpreters specializing in Vietnamese, we recruited research collaborators throughout Japan. As a result of the interview survey, the following five categories were identified as the main reasons for discontinuation of treatment: “financial burden of TB treatment”, “burden of going to the hospital”, “knowledge of TB not properly understood”, “prejudiced view of TB treatment by others”, and “no one close to them to support TB treatment”. These were the main reasons for the discontinuation of treatment [29].

#### 2.4.3. Literature Review on Treatment Interruption in Vietnamese Patients with TB

Using PubMed and ICHUSHI, we searched for literature on the risk of treatment interruptions among Vietnamese patients with TB and on medical visits by foreign residents, using keywords such as “tuberculosis”, and “treatment” interruption”. We found and analyzed 49 studies that met our objectives. Studies related to medical care for foreign residents (treatment, access, relationship with medical personnel, etc.), language issues, such as the need for interpreters, and the living conditions for Vietnamese technical interns and foreign students were the most frequently found. Based on these studies, a draft of risk assessment items for Vietnamese patients with TB was developed [30].

#### 2.4.4. Risk Assessment Items and Interviews with TB Experts

After receiving feedback from the medical interpreters and developing the risk assessment items based on our literature review, a survey of TB experts was conducted to determine the validity of the devised risk assessment items and the wording of the items. The TB experts include Vietnamese doctors specializing in respiratory medicine who have treated many Vietnamese patients with TB, TB nursing researchers, and researchers who support foreign patients with TB. After receiving their expert opinions, we finalized a draft risk assessment items form consisting of 28 items in seven categories [30].

### 2.5. Data Collection

To increase the likelihood that the respondents would meet the selection criteria for research collaborators, the survey was conducted at health centers throughout Japan, with the Vietnamese population accounting for at least 10% of the total foreign population, or with a Vietnamese population of at least approximately 2000 people [31]. Consequently, 48 health centers nationwide were selected for the study. These health centers were contacted via telephone to explain the study purpose to the person in charge of tuberculosis services and identify whether any research collaborators met the selection criteria. If consent was obtained, a letter of request was sent to them to request their cooperation.

The survey was conducted via a questionnaire sent by post. The participants were asked to complete the questionnaire and return it by post, along with a letter requesting their cooperation, the questionnaire itself, and a self-addressed stamped envelope. The survey was conducted three times, and the questionnaire was a self-administered anonymous survey. The survey asked for the research participants’ basic attributes (their age, occupation, work history, experience in TB work with the Vietnamese population, number of TB cases handled, and TB research experience) as well as their opinions about the validity of the draft risk assessment items. The collaborators were asked to respond to 28 items in seven categories. Regarding content validity, each risk assessment item was rated on a 9-point scale, ranging from “not at all appropriate” to “very appropriate”.

The first survey response was returned, and because there was a possibility that the intent of the risk assessment items was not correctly conveyed to the collaborator regarding the item that was rated low as a risk assessment item, we carefully added the reason that it was a risk item in the next survey request so that the item could be rated more correctly.

The surface validity of the survey was free writing, and for each classification and each risk assessment item, respondents were asked to freely write about the following: 1. The accuracy of the intended content; 2. The appropriateness of the textual expression; 3. The similarity between items; 4. Possible items other than the presented risk assessment items; and their opinions, if necessary. For each classification and risk assessment item that was modified based on the comments noted in the responses to reflect them in the next survey, efforts were made to write a detailed and clear explanation of the reasons for the modifications, which were then repeated.

In each of the second and third surveys, the respondents were asked to rate all risk assessment items in the questionnaire.

Regarding the dropout of research collaborators, a two-week period was set to prevent forgetting to respond, while considering the length of the response period and to avoid placing an excessive burden on them. For the dropouts at this time, no reminders were sent out, as the research collaborators’ workplaces contacted them about participation.

The study period was from January to March 2023.

### 2.6. Analysis Method

The content validity index (hereafter, CVI) was calculated using the content validity quantification method [32,33]. The validity of each item (hereafter, item CVI) was rated on a nine-point scale, with a score of one to four being “not valid”, a score of five—the median of the nine-point scale—being “difficult to judge”, and a score of six or higher being “valid”.

The consensus criteria were a median score of at least seven points and a CVI of at least 0.78 for each risk assessment item. We calculated the item CVI for each risk assessment item, and items with low scores were considered for removal, modification, or integration based on the opinions of the participants while considering the validity of the content. In addition, along with modifying the risk assessment items, the classifications were modified, and the validity of the classification of each risk assessment item was examined repeatedly.

Regarding superficial validity, we used a qualitative analysis method to synthesize similar opinions within each item, and then organized the opinions. The organized opinions were repeatedly examined by researchers for the accuracy of the intended content of each risk assessment item, duplication of meaning, and appropriateness of expression, and the items were revised, added, deleted, or integrated.

### 2.7. Ethical Considerations

This study was approved by “the Ethics Review Committee on Research Involving Human Subjects” (21-048-04) of Nihon Fukushi University. Consent was obtained from all research collaborators. They were informed of the purpose of the study and the voluntary nature of their participation, that they could withdraw their participation at any time, and that the survey would be anonymous. There would also be no detriment if they withdrew from participation.

## 3. Results

### 3.1. Characteristics of Research Collaborators (Table 1)

We surveyed 15 collaborators, including 13 public health nurses and 2 healthcare workers, to validate the key risk factors affecting treatment interruption for Vietnamese patients with TB in Japan. All the research collaborators obtained through the selection criteria had experience of being the key person in charge of providing support to multiple Vietnamese-born patients with TB using the Japanese version of DOTS; had been engaged in providing care and support to a single patient for at least six months (the standard treatment period for TB); and had gained a good understanding of Vietnamese culture, lifestyle, and views on health during that time. Of these participants, 10 worked at the largest health centers, with populations ranging from 500,000 to 1 million. The two non-public health nurses were experienced in dealing with Vietnamese patients with TB, were in charge of patient management at health centers, and had a history of TB research.

**Table 1:** Summary of research collaborators.

		Number of People	(%)
Date	Under 30	1	6.7
	30–39 years old	3	20.0
	40–49 years old	7	46.7
	50–59 years old	4	26.7
Population size of their health center	200,000 to 500,000	4	26.7
	500,000 to 1,000,000	10	66.7
	More than 1 million	1	6.7
Type of occupation	Public health nurse	13	86.7
	Other	2	13.3
Profession experience	5 years	1	6.7
	6–10 years	4	26.7
	11–15 years	3	20.0
	16–20 years	2	13.3
	More than 20 years	5	33.3
Tuberculosis work history	About 3 years	7	46.7
	3–5 years	1	6.7
	6–10 years	7	46.7
Number of Vietnamese patients with TB handled	2 people	4	26.7
	3 people	4	26.7
	4 people	3	20.0
	5 or more	3	20.0
	Unknown	1	6.7
Tuberculosis research history	Nothing	12	80.0
	1–4 years	1	6.7
	5 to 9 years	2	13.3

Examining the participants’ experience in managing patients with TB, 7 of the individuals indicated that they had about 6–10 years of experience and another 7 stated they had about 3 years of TB work experience, with one respondent indicating 3–5 years of experience. In response to the question about TB research, three participants had a history of TB research, including two with more than five years of research experience.

### 3.2. Confirming the Draft Risk Assessment Items

#### 3.2.1. Validity of the First Survey for Risk Assessment Items Among Vietnamese Patients with TB in Japan

All 15 collaborators (survey participants) completed the questionnaire (Table 2). Based on the responses, for classification purposes, “Social and Life Background” was split into “Life Background” and “Social Background”. Life background included food, clothing, shelter, and rhythm of life, while social background included employment, access to hospitals, social insurance, and collaborators. “Understanding Tuberculosis” and “Acceptance of Tuberculosis” were combined into one category from the perspective of how patients perceived their disease. “Difficulty communicating in different languages” was categorized as “Language and cultural risk”, and language and other cultural differences were lumped together.

Seven risk assessment items received an item CVI of less than 0.78. Under the category “Medical condition”, the item “Complications (e.g., diabetes, immunosuppressive drugs, dialysis, a disease with corticosteroid use)” had a median score of six and an item CVI of 0.53 and were thus eligible for removal. However, because eight of the research collaborators indicated that the appearance of complications is associated with worsening TB and is a valid risk factor, it was decided to not delete it but to revise the description by adding “cancer and HIV” based on the research collaborators’ stated opinions. Regarding the item ”History of completed TB treatment in the past”, if the patient relapsed despite previous treatment, there would be a possibility of treatment discontinuation due to patient demotivation; but the item CVI did not meet the consensus threshold, in addition to the opinion that only a few patients in Vietnam have a history of treatment; hence, the questionnaire was partially revised, the question wording was partially modified by integrating it with “Risk assessment items 12, 14, 15, 16, and 22”—which met the consensus threshold—but the wordings were modified based on the opinions that these should be more easily understood.

The classification of four items, (25, 26, 27 and 28) in “Characteristics and specifics of Vietnamese patients with TB”, did not meet the consensus threshold. However, in terms of overall scores, their median scores were seven for all participants, which met the validity assessment criterion of six. Since more than 9 out of the 15 collaborators rated the items as six or higher, we decided to examine each risk assessment item again. “Low awareness of self-care” was shown to be difficult to understand because of its vague language. Thus, the wording was changed to convey that the respondents were more interested in their own interests than in self-care and that treatment, which should be a priority, was put on the back burner.

Item 26 was changed to clarify the content of the risk assessment item because the wording was similar to “poor acceptance of DOTS” and “avoid contact with DOTS partners”. In this item, the Vietnamese cultural background of valuing conformity with the surroundings and the consciousness of isolating people with infectious diseases remain deeply rooted in society, and the risk is that people will avoid contact with others for fear of being discriminated against by those around them. Part of the item “poor acceptance of DOTS” was merged with “I am concerned about the evaluation of tuberculosis treatment by others” in the item “Because of the similarity in semantic content”, and changed to “I want to hide the fact that I am receiving TB treatment from those around me”. On the other hand, “avoid contact with DOTS partners” was changed to “Show an attitude of avoiding DOTS partners”, including the implications of Vietnamese culture, which values courtesy and consideration for others. In addition, since items 27 and 28 were related to the purpose of the patient’s visit to Japan, both items were integrated into one.

We explained these changes and the reasons for the same and asked them to evaluate all items in the next survey.

#### 3.2.2. Validity of the Second Survey for Risk Assessment Items Among Vietnamese Patients with TB in Japan

We mailed a second survey to the same 15 participants and received responses from 14 of the 15 individuals. Based on the responses, the item CVI improved. All four items in classification “The trends observed in Vietnamese patients with TB” improved, and all but one met the consensus threshold. Only one item “Complications”, did not meet the item CVI of 0.78. The opinion expressed regarding this was that poor control of complications, rather than the presence or absence of complications, would be associated with the risk of treatment interruption. The wording was then revised in response to these comments. Furthermore, in the opinion of the study collaborators, an expired Visa was added as a new risk assessment item because it is associated with the risk of treatment interruption (Table 3).

Regarding the wording of the risk assessment item, the wording was revised because there were several similar opinions in the following two items (items 6 and 24). The item “Do not own a residence” does not mean homeless, but rather “does not have a specific residence and moves from one friend’s house to another”; thus, “No residence of their own, moving from one friend’s house to another” was finalized. For item “Shows an attitude of avoiding contact with DOTS partners”, we used “shows an attitude of avoiding contact with DOTS partners (e.g., not being able to contact them)” to improve readability.

For other items, we examined whether the wording was specific, easy to understand, and appropriate, referring to the opinions of the research collaborators. The item “Irregular rhythm of life” and “Inadequate diet and nutrition, frequently missing meals” were merged into item 9 because the contents were similar. In addition, items 16 and 17 were integrated into “Insufficient understanding of treatment policies, treatment details, and medication side effects” because of the lack of understanding of the contents of tuberculosis treatment. The item “Physical or mental (including memory, cognition, etc.) disability” is suitable as a risk assessment item for tuberculosis patients in general, but was removed from the risk list because it seemed that only a few Vietnamese tuberculosis patients who entered the country as technical intern trainees or international students had disabilities.

As a result of reviewing the categories based on these revisions, they were organized into six categories. As in the previous survey, we explained the above changes and the reasons for those changes and asked them to evaluate all items in the next survey.

#### 3.2.3. Validity of Third Survey for Risk Assessment Items Among Vietnamese Patients with TB in Japan (Table 4)

After an inter-researcher discussion, the factors shown in Table 4 were mailed again to the same 15 collaborators as in the first round, with responses received from 13 participants. Regarding each risk assessment item in Table 4, we asked whether any of the items were inappropriate or required modification. No additional revisions were indicated for any risk assessment items. Thus, we were able to ensure the validity of the risk assessment form in Table 4.

Finally, the categories and risk assessment items were changed as follows: five categories, one new risk item, one deleted risk item, four items were integrated between categories, and seven items were changed in wording (Table 5).

## 4. Discussion

### 4.1. Validity of the Survey Design Based on the Delphi Method

This study was conducted to develop risk assessment items to be used during patient assessment in the DOTS as part of the support for Vietnamese patients with TB, the largest and fastest-growing group of patients with TB in Japan. Draft risk assessment items extracted from the literature were reviewed by four Vietnamese TB experts to improve the accuracy of the factors in the survey. The 15 collaborators for this study were recruited from across the country, the largest number of those collaborators had more than 20 years of work experience, and two-thirds of them had more than 10 years of work experience. The condition for selecting collaborators was that they had experience in handling DOTS for multiple Vietnamese patients with TB, and based on these facts, we believe that the quality of the study collaborators was sufficiently assured. On the other hand, in terms of the number of collaborators in this study, some studies were conducted by 19 subjects in studies using the Delphi method, and some studies were conducted by 5 subjects in studies using the I-CVI for content validity [34,35]. The accuracy of the results is enhanced by multiple administrations by high-quality collaborators. In this study, the survey was conducted three times by high-quality collaborators, and finally, all collaborators agreed on the results. Therefore, we believe that the risk assessment items developed in this study for Vietnamese patients with TB were validated.

### 4.2. Classifications and Items for Risk Assessment Items of Treatment Interruption Among Vietnamese Patients with TB Under DOTS in Japan

The final risk assessment items we developed for Vietnamese patients with TB include 24 items in five categories. In the “Physical characteristics” category, younger foreign patients with TB (15–35 years old) currently account for 73% of all TB patients; this is also true for Vietnamese patients with TB, with the highest percentage living in Japan for less than five years, including technical interns and foreign students [6]. Among the five categories, “Living background in Japan” can capture the potential poverty and instability in Japan. Although many Vietnamese come to Japan to enrich their lives in their home countries, life in Japan is difficult, and receiving TB treatment is a heavy financial burden. In Japan, a medical system covers a certain percentage of such patients’ TB medical expenses through public funds. Although the co-payment percentage is reduced, patients from low-income backgrounds need to pay not only medical expenses but also transportation costs to see a doctor, making TB treatment a potentially life-threatening situation for economically impoverished patients [30,36,37,38]. We believe that financial burden is a major risk factor for treatment interruption. In category 3, “treatment environment”, a lack of understanding of treatment in the workplace was also cited as a risk. Patients with TB are also concerned about the impact on their company’s reputation. International students often work part-time jobs and fear that they will be fired by their part-time employers if they take time off work. Similarly, technical intern trainees hesitate to take time off work because they do not want to give a bad impression to their supervisors or employers. In Japan, workers have the right to take paid leave, but they fear that taking a break for hospital visits will give their employer or school a bad impression. The Vietnamese culture encourages people to be serious, diligent, and respectful of others; thus, some Vietnamese individuals in Japan may feel uneasy about asserting themselves in such a way that would interfere with their employers [29]. Difficulties with hospital visits also involve language understanding. Without the ability to read Japanese, the burden on the patient is even greater [39]. In such treatment environments, patients need to have a reliable medication companion close at hand. Even Vietnamese individuals who came to Japan as technical interns with others might have strong prejudice against TB and might keep their distance when they learn that these individuals have developed TB [40,41,42]. In such cases, the support of DOTS partners is essential. In the “Japanese version of DOTS”, we worked with individuals supporting DOTS in the community to eliminate TB and provide medical support aimed at complete treatment of patients. It is also necessary for DOTS partners to work together to improve the patient care environment, including helping patients identify trusted associates and collaborators.

Category 4 “Understanding tuberculosis and acceptance of the disease” includes a risk factor based on the patients’ incorrect understanding of TB. Owing to incorrect knowledge of TB in their home countries, some individuals may have developed misconceptions about TB, are unable to accept the TB diagnosis, and are unwilling to receive treatment based on medical evidence. One main reason for this may be the language barrier, implying that accurate knowledge about TB is not communicated to patients. Additionally, the risk items 21, 22, and 23 of category 5, “cultural and value tendencies”, are considered related to Vietnamese culture, and caused by individual patients’ lack of accurate TB knowledge. Patients may want to hide their TB to avoid alienating others, they may show an attitude of not wanting to be contacted by DOTS partners, or they may put off treatment in favor of immediate benefits rather than a complete cure for TB [29]. In other words, due to the language barrier, the knowledge regarding TB treatment is not accurately conveyed, and patients remain uneducated or misunderstood. Some characteristics of the Vietnamese culture, such as “goodwill” and “concern for others”, are considered to be further factors promoting the discontinuation of TB treatment [43]. To avoid these risks, careful explanation in their native language is needed through a medical interpreter. Many foreign students and technical intern trainees come to Japan with a certain degree of Japanese language education in their home countries; thus, it is unlikely that they have no understanding at all. As for technical intern trainees, there are often Vietnamese interpreters in the workplace who can converse at the level of daily life, so there is no hindrance to living. However, interpreters with limited medical knowledge are unable to accurately convey medical terminology and may take the convenience of the workplace for granted, leading to situations in which patients misunderstand the information [29]. This creates the possibility for patients to misinterpret information. Communicating accurate information is very important to avoid treatment interruptions; DOTS personnel should provide accurate knowledge via medical interpreters during DOTS, with this information repeated many times during the treatment period.

### 4.3. Implications for Nursing Practice Derived from DOTS Care Based on the Individualization of Assessment Items

The risk assessment items for Vietnamese patients with TB obtained in this study were grouped into five categories, including items related to the disease, as well as the environment surrounding the patient after arriving in Japan, and risks due to the patient’s cross-cultural background. Vietnamese patients with TB were shown to have more risk assessment items to focus on than Japanese patients. To continue the treatment of this group, it is necessary to provide patient care that focuses even more on the risks in the patient’s medical-care life than Japanese patients. The results of this study listed the categories of “life background during residence”, “treatment environment”, and “cultural/value trends”, showing that Vietnamese patients with TB living in the community were at higher risk than Japanese patients, which makes care focusing on the treatment of Vietnamese patients with TB living in the community indispensable [29]. DOTS plans prepared at public health centers are mainly developed by public health nurses. It is necessary to develop a DOTS plan that comprehensively perceives the patient from a nursing perspective and—as a nursing professional who is familiar with the actual conditions in the community—to promote collaboration with multiple community-based institutions and professionals, thereby enabling the patient to continue treatment. Finding a DOTS partner for the patient and adjusting and improving the patient’s care environment so that the patient can continue to receive treatment in the community should also be considered as part of nursing care.

Although the risk assessment items developed in this study are tools to guide successful TB treatment, they were developed from a nursing perspective based on the holistic view of the patient, including the patient’s cultural background, living environment during residency, and treatment environment. It is important to consider these items individually to eliminate any risk identified in the assessment. In other words, it is important not to simply count the risk assessment items of Vietnamese patients with tuberculosis, but to take a comprehensive view of the patients, thereby eliminating the relevant items together with the patients.

The risk assessment items developed in this study are limited to Vietnamese patients with TB living in Japan; so, they are not universal, and the risk items are likely to differ depending on the culture, economy, and social situation of other countries. However, based on the results of this study, we emphasize the importance of providing support for patients who develop tuberculosis while living abroad, considering their upbringing and living environment.

### 4.4. Limitations of the Study and Future Challenges

This study requested that health centers with large Vietnamese populations throughout Japan cooperate with our survey of mid-level public health nurses and other practitioners who had experiences in dealing with DOTS for Vietnamese patients with TB. As the study was conducted in the aftermath of COVID-19, the number of health centers that could participate in the survey was limited. However, as people’s fears about COVID-19 subside, it is expected that the number of Vietnamese TB patients visiting Japan will increase.

In addition, although this study targeted public health centers with a high incidence of Vietnamese patients with TB, the risk factors may change slightly in areas with only a few Vietnamese patients with TB, such as rural areas. As the risk factors will differ depending on the characteristics of each area, the limitation of this study is that the results of this study will need to be arranged and utilized for each public health center.

As future research, it is necessary to devise a research design that uses this risk assessment item in actual Japanese DOTS, and to carefully verify whether it accurately reflects the characteristics of Vietnamese patients with TB receiving treatment in Japan. We also aim to conduct empirical research by collecting data from actual Vietnamese tuberculosis patients, specifically those with experience in treatment interruption. Furthermore, as a risk assessment form for Filipino tuberculosis patients living in Japan has already been developed in the previous research, a demonstration study of this research in the future may be conducted and compared with the Filipino version [44].

## 5. Conclusions

The risk assessment items we developed for Vietnamese patients with TB include 24 risk assessment items in five categories: “Physical characteristics”, “Background of life during residence”, “Treatment environment”, “Understanding tuberculosis and acceptance of the disease”, and “Cultural and value trends”. The content is multifaceted, including living conditions in Japan, Vietnamese culture, and the values held by patients. Specifically, it demonstrates the risk of treatment interruption in Vietnamese patients with TB from a Japanese perspective. We believe that this will also be useful for patient support in DOTS for Vietnamese patients with TB living in various countries worldwide.

## Figures and Tables

**Table 2:** The validity of the first survey for risk assessment items among Vietnamese patients with TB in Japan.

Classification.	Number/Risk Assessment Item		Median	Number of People over 6 Points	CVI: over 0.78
1. Medical condition	1	Apparent side effects from anti-tuberculosis drugs	9	13	0.87
	2	Complications (e.g., diabetes, immunosuppressive drugs, dialysis, disease with corticosteroid use)	6	8	0.53
	3	Drug resistance to anti-tuberculosis drugs	8	13	0.87
	4	History of treatment interruption in the past	9	14	0.93
	5	History of completed TB treatment in the past	7	9	0.60
2. Social and Life Background	6	Financially needy in terms of livelihood	9	14	0.93
	7	Do not own a residence	9	13	0.87
	8	Repetitive relocation to the region for good income and good conditions	9	14	0.93
	9	Irregular rhythm of life	9	14	0.93
	10	Inadequate diet and nutrition, frequently missing meals	9	14	0.93
	11	Have multiple jobs or work long hours	9	14	0.93
	12	Insufficient understanding in the workplace about receiving medical treatment	9	15	1.00
	13	There are obstacles or difficulties in accessing hospital visits	9	15	1.00
	14	Not enrolled in health insurance	9	13	0.87
	15	No collaborator during recuperation	9	15	1.00
3. Understanding Tuberculosis	16	Insufficient understanding of the characteristics of TB	9	12	0.80
	17	Insufficient understanding of treatment details, etc.	9	13	0.87
	18	Insufficient understanding of the side effects of medications	9	13	0.87
4. Acceptance of Tuberculosis	19	Not accepting that they have TB	9	13	0.87
	20	Concerned about others’ evaluation of their TB treatment	7	9	0.60
5. Mental and Physical Condition	21	Physical or mental (including memory, cognition, etc.) disability	9	13	0.87
	22	Alcohol and drug dependence and smoking (or passive smoking)	9	13	0.87
6. Difficulty communicating in different languages	23	Difficulty understanding and communicating in Japanese (daily conversation level or lower)	9	15	1.00
7. Characteristics of purposefully arrived Vietnamese patients with Tuberculosis in Japan.	24	Not interviewed in their native language by a medical interpreter from a public institution	9	14	0.93
	25	Low awareness of self-care	7	9	0.60
	26	The DOTS * is poorly accepted and avoids contact with DOTS partners	7	10	0.67
	27	Feeling that continued treatment would interfere with the purpose of coming to Japan	7	9	0.60
	28	No goal to complete treatment	7	10	0.67

* Directly Observed Treatment short-course.

**Table 3:** The validity of the second survey for risk assessment items among Vietnamese patients with TB in Japan.

Classification.	Changes, etc.	Number/Risk Assessment Item		Median	Number of People over 6 Points	CVI: over 0.78
1. Medical Condition		1	Apparent side effects from anti-tuberculosis drugs	9	14	1.00
	revision	2	Complications (diabetes, immunosuppressive drugs, dialysis, corticosteroid use, cancer, HIV, etc.)	9	10	0.71
		3	Drug resistance to anti-tuberculosis drugs	9	14	1.00
	revision	4	History of tuberculosis treatment (including history of interruption)	9	13	0.93
2. Life Background		5	Financially needy in terms of livelihood	9	14	1.00
		6	Do not own a residence	9	12	0.86
		7	Repetitive relocation to the region for good income and good conditions	9	12	0.86
		8	Irregular rhythm of life	9	13	0.93
		9	Inadequate diet and nutrition, frequently missing meals	9	14	1.00
		10	Have multiple jobs or working long hours	9	14	1.00
3. Social Background	revision	11	Insufficient understanding in the workplace about receiving TB treatment	9	13	0.93
		12	Obstacles or difficulties in accessing hospital visits	9	13	0.93
	revision	13	Not enrolled in health insurance or arrears of premiums	9	14	1.00
	revision	14	Lack of a trusted collaborator for recuperation	9	13	0.93
4. Understanding Tuberculosis and Disease Acceptance	revision	15	Insufficient understanding of the disease and its characteristics of TB	9	14	1.00
		16	Insufficient understanding of treatment details, etc.	9	14	1.00
		17	Insufficient understanding of the side effects of medications	9	14	1.00
		18	Not accepting that they have TB	9	14	1.00
5. Mental and Physical Condition		19	Physical or mental (including memory, cognition, etc.) disability	9	13	0.93
	revision	20	Dependence on luxury items (alcohol, tobacco) or drugs	9	13	0.93
6. Language and Cultural Differences		21	“Difficulty in understanding and communicating in Japanese (Daily conversation level or lower)”	9	14	1.00
		22	Not interviewed in their native language by a medical interpreter from a public institution	9	13	0.93
7. Characteristics of purposefully arrived Vietnamese patients with Tuberculosis in Japan.	change	23	Wants to hide from others that they are undergoing TB treatment	9	14	1.00
	change	24	Demonstrates an attitude of avoiding contact with DOTS partners	9	13	0.93
	change	25	Easier to focus on other things rather than one’s self-care	7	11	0.79
	change	26	They believe that tuberculosis treatment hinders their own purpose in coming to Japan	9	12	0.86

**Table 4:** The validity of the third survey for risk assessment items among Vietnamese patients with TB in Japan.

Classification.	Changes, etc.	Number/Risk Assessment Item	
1. Physical Characteristics		1	Apparent side effects from anti-tuberculosis drugs
		2	Poorly controlled complications
		3	Drug resistance to anti-tuberculosis drugs
		4	Dependence on luxury items (alcohol, tobacco) or drugs
2. Background of life during Residence		5	Financially needy in terms of livelihood
	revision	6	No residence of their own, moving from one friend’s house to another
		7	Repetitive relocation to the region for good income and good conditions
	add (e.g., annex)	8	Visa Expiration
	integration	9	Inadequate diet and nutrition, frequently missing meals
		10	Have multiple jobs or working long hours
3. Treatment Environment		11	Insufficient understanding in the workplace about receiving TB treatment
		12	Not enrolled in health insurance or arrears of premiums
		13	There are obstacles or difficulties in accessing hospital visits
		14	Lack of a trusted recuperate collaborator
4. Understanding Tuberculosis and Disease Acceptance		15	Insufficient understanding of the disease and its characteristics of TB
	integration	16	Insufficient understanding of treatment policies, treatment details, and medication side effects
		17	Not interviewed in their native language by a medical interpreter from a public institution
		18	Not accepting that they have TB
		19	History of tuberculosis treatment (including history of interruption)
5. Vietnamese Cultural and Value trends		20	Difficulty in verbal comprehension and communication (daily conversation or less)
		21	Wants to hide from others that they are undergoing TB treatment
	revision	22	Attitude of avoidance of contact with DOTS partners (e.g., inability to contact them)
		23	Easier to focus on other things rather than one’s self-care
		24	They believe that tuberculosis treatment hinders their purpose in coming to Japan

**Table 5:** Confirming the draft risk assessment items.

Classification				
Type of change	No	Draft classification and risk item	Result of qualitative content as of the 3rd (final result)	
Correction of notation	1	Medical condition	1	Physical Characteristics
Separation	2	Social and Life Background	2	Background of life during Residence
			3	Therapeutic Environment
Integration	3 4 5	Understanding Tuberculosis Acceptance of Tuberculosis Mental and Physical Condition	4	Understanding Tuberculosis and Disease Acceptance
	6 7	Difficulty communicating in different languages Characteristics of purposefully arrived Vietnamese patients with Tuberculosis in Japan.	5	Vietnamese Cultural and Value trends
Risk Items				
Type of change	No	Draft classification and risk item	Result of qualitative content as of the3rd (final result)	
Addition			8	Visa Expiration
Deletion	21	Physical or mental (including memory, cognition, etc.) disability		
Integration	4 5	History of treatment interruption in the past History of completed TB treatment in the past	19	History of tuberculosis treatment (including history of interruption)
	9 10	Irregular rhythm of life Inadequate diet and nutrition, frequently missing meals	9	Inadequate diet and nutrition, frequently missing meals
	17 18	Insufficient understanding of treatment details, etc. Insufficient understanding of the side effects of medications	16	Insufficient understanding of treatment policies, treatment details, and medication side effects
	27 28	Feeling that continued treatment would interfere with the purpose of coming to Japan No goal to complete treatment	24	They believe that tuberculosis treatment hinders their purpose in coming to Japan
Correction of notation	2	Complications (e.g., diabetes, immunosuppressive drugs, dialysis, disease with corticosteroid use)	2	Poorly controlled complications
	7	Do not own a residence	6	No residence of their own, moving from one friend’s house to another
	14	Not enrolled in health insurance	12	Not enrolled in health insurance or in arrears of premiums
	16	Insufficient understanding of the characteristics of TB	15	Insufficient understanding of the disease and its characteristics of TB
	20	Concerned about others’ evaluation of their TB treatment	21	Wants to hide from others that they are undergoing TB treatment
	25	Low awareness of self-care	23	Easier to focus on other things rather than one’s own self-care
	26	The DOTS * is poorly accepted and avoids contact with DOTS partners	22	Attitude of avoidance of contact with DOTS * partners (e.g., inability to contact them)

* Directly Observed Treatment short-course.

## Data Availability

The data collected in this study are not available to the public for reasons of informed consent and confidentiality but may be obtained from the corresponding author upon reasonable request.

## Public Involvement Statement

There was no public involvement in any aspect of this research.

## Guidelines and Standards Statement

This manuscript was drafted based on Research guidelines for the Delphi survey technique. (https://onlinelibrary.wiley.com/doi/10.1046/j.1365-2648.2000.t01-1-01567.x) (accessed on 18 March 2024).
